# Perillaldehyde inhibits bone metastasis and receptor activator of nuclear factor-κB ligand (RANKL) signaling-induced osteoclastogenesis in prostate cancer cell lines

**DOI:** 10.1080/21655979.2021.2001237

**Published:** 2022-01-26

**Authors:** Zhuoyuan Lin, Sheng Huang, Xitao LingHu, Yixiao Wang, Bin Wang, Shaowen Zhong, Shangyan Xie, Xiaohong Xu, Aorigele Yu, Atsushi Nagai, Yuta Kobayashi, Qingde Wa, Shuai Huang

**Affiliations:** a Department of Urology, The Second Affiliated Hospital of Guangzhou Medical University, Guangzhou, China; b Department of Orthopedics, The First Affiliated Hospital of Nanchang University, Nanchang, China; c Department of Orthopedics, The Second Affiliated Hospital of Zunyi Medical University, Zunyi, China; d Graduated School of Medicine, Shimane University, Izumo, Japan

## Abstract

Perillaldehyde (PAH), one of the active ingredients of the traditional Chinese medicine (TCM) plant *Perilla frutescen*s, is widely used and exerts crucial anti-cancer activities. The aim of current study is to illustrate the potential mechanisms of PAH-mediated regulation of bone metastasis and osteoclastogenesis in prostate cancer (PCa) cell lines. Effects of PAH on proliferation, invasion and migration of PC-3 cells were assessed with the Cell Counting Kit-8 (CCK-8) assay and Transwell assays, respectively. Effects of PAH on stem cell characteristics of PC-3 cells were evaluated by cell-matrix adhesion assay, colony formation assay, spheroid formation assay, as well as western blot . The anti-metastasis and anti-osteoclastogenesis activity of PAH in RAW264.7 cells was examined by osteoclast differentiation assay and western blot. The protein levels of CD133 and CD44 in PC-3 cells and the activity of nuclear factor kappa B (NF-κB) signaling pathway in RAW264.7 cells were measured by western blot. PAH suppressed proliferation, invasion and migration of PC-3 cells, prevented stem cell characteristics including cell-matrix adhesion, colony formation, spheroid formation as well as CD133 and CD44 expression. PAH inhibited bone metastasis and osteoclastogenesis via repressing the activation of NF-κB pathway as well as (RANKL) – and cancer cell-induced osteoclastogenesis in PCa cells. These findings suggested the potential therapeutic effects of PAH on the metastasis of patients with PCa.

## Introduction

Prostate cancer (PCa) remains one of the most frequent cancer types in males and the 3^rd^ most frequent cause of cancer-related mortality in males, with ≥ 161,000 new cases in the United States in 2017[1]. One of the most frequent complications of PCa is bone metastasis, which is observed in 70% of patients with advanced PCa. Once bone metastases occur, the patients would become incurable and exhibit a high mortality [1–4].

Bone metastases cause numerous complications, including hypercalcemia, pathologic bone fractures and bone pain, *etc*[5]. As acknowledged, there are four essential metastatic steps for metastatic cells, ie, detachment, migration, invasion and adhesion[6].

Osteoclastogenesis is usually associated with PCa by activating the receptor activator of nuclear factor-κB (RANK) ligand (RANKL) signaling pathway [7,8]. Previous researches have shown that RANK, RANKL, NF-κB and inhibitor of nuclear factor kappa B (IκB) kinase (IKK) play important roles in the differentiation of osteoclasts [9,10]. NF-κB which is a heterodimer protein composed of p65 and p50, is activated during osteoclast differentiation. IKKα and IKKβ are two kinase subunits of IKK, an essential activator for NF-κB. The activation of RANKL pathway prompts the activation of diverse downstream pathways required for osteoclast development. Novel drugs that target RANKL pathway in osteoclasts could be effective treatments for bone metastases in PCa[11]. Pyrophosphate analogs, bisphosphonates and human monoclonal antibody to RANKL, denosumab are widely used to relieve the symptoms of patients with bone metastases[12].

Numerous studies of products derived from plants of TCM have been carried out to evaluate their effects in the patients with metastatic PCa[13]. Perilla, also known as red Perilla, labiaceae annual herbs. The whole body of Perilla has different pharmacological effects and its extract oil also has good research value[14]. Monoterpenes, triterpenes, flavonoids, phenylpropyl and phenols were the main active compounds isolated from Perilla Perilla. It has significant effects on lowering blood lipid, anti-allergy, anti-tumor, anti-atherosclerosis and anti-oxidation, etc[15]., which has attracted the attention of researchers at home and abroad and the favor of consumers. Perilla Perilla is rich in phenolic compounds. The oil extracted from Perilla Perilla is detected to contain 65 kinds of polyphenolic compounds, among which the largest content is perilaldehyde, perilone, dehydroperilone, etc[16]., which has various active functions such as anti-tumor, anti-cardiovascular disease, antibacterial and anti-oxidation. The antioxidant activity of Perilla is an important property. Emi Saita et al [17]. found that Perilla is rich in polyphenols, which can significantly inhibit azo free radicals and low-density lipoprotein oxidation induced by endothelial cells, and can significantly increase the expression levels of antioxidant protein and mRNA in endothelial cells. Perilla aldehyde (PAH), as an extract of Perilla Perilla, has strong anti-microbial[18], anti-inflammatory[19], anti-oxidant[20], and anti-cancer activities[21]. At the same time, perilaldehyde also has a good role in the treatment of depression [22] and repair DNA damage[23]. However, it has not been reported about the potential ability of PAH in inhibiting PCa bone metastasis.

The current study aimed to identify the ability of PAH in modulating the activities of human bone metastatic PCa cells, RANKL- and cancer cell-induced osteoclastogenesis. We found that PAH suppressed proliferation, invasion and migration of PC-3 cells. Similarly, PAH were observed to inhibited the cell-matrix adhesion, colony formation, spheroid formation and cancer cell- induced osteoclastogenesis of PC-3 cells in vitro. All of these effects may be attributed to activate the NF-κB pathway and receptor activator of nuclear factor-κB ligand. These findings revealed the potential therapeutic effects of PAH on the metastasis of patients with PCa.

## Materials and methods

### Reagents and antibodies

PAH was purchased from Sigma-Aldrich (St. Louis, MO, USA). The antibodies were used as following: anti-CD133 and anti-CD44 antibodies (Abcam, Cambridge, UK); anti-GAPDH, anti-p65 and anti-p50 antibodies (Santa Cruz Biotechnology, Dallas, TX, USA); and antibodies againstIKKα, IKKβ and RANKL (Cell Signaling Technology, Danvers, MA, USA).

### Cell lines

PC-3 cells and RAW264.7 cells (ATCC, Manassas, VA, USA) were grown in DMEM containing 10% FBS and 1% antibiotics, at 37°C, with 5% CO_2_ in a humid air environment. RAW264.7 cells could differentiate into functional tartrate resistant acid phosphatase (TRAP)-positive osteoclasts after incubation with soluble RANKL.

### Cell proliferation assay

The cell proliferation assay in PC-3 cells was evaluated by CCK-8 assay kit (Dojindo, Kumamoto, Japan). Briefly, PC-3 cells were grown for 36 h in 96-well plates with increasing concentrations of PAH (0, 0.25, 0.5, 1, 2 μM). The absorbance at 450 nm was recorded by ELx808 microplate reader (Bio-tek, Winooski, VT, USA). Five random wells were selected for each concentration.

### Transwell invasion and migration assay

The invasion and migration assays were performed as previously described[24]. As for the invasion assay, PC-3 cells were pre-treated with PAH (0, 0.25, 0.5, 1 μM) for 24 h, prior to being plated onto a new plate and further cultured in serum-free medium. Subsequently,100 μL cells (density 5 × 10^4^ cells/mL) in serum-free DMEM were added into the upper chamber of Transwell which is pre-coated with Matrigel (BD Biosciences, Billerica, MA, USA), while 600 μL of DMEM with 10% FBS were added into the lower chamber. At 24 h after incubation, some cells were penetrated from the upper chamber through the membrane to the lower chamber, which were fixed by 4% paraformaldehyde (PFA) and stained by 0.1% crystal violet. Thereafter, the penetrated cell numbers were quantified from five randomly selected x100 microscopic areas. For migration assay, it was performed the same as invasion assay, except for the use of Matrigel-coated membrane.

### Cell-matrix adhesion assay

The cell-matrix adhesion assay was modified from a previous protocol[25]. After incubation for 24 h at 95% confluence, PC-3 cells were pre-treated by PAH (0, 0.25, 0.5, 1 μM) for 24 h prior to being plated on 96-well plates coated with fibronectin (50 g/ml) and cultured at 37°C for another 1 h. Followed with fixation by 4% PFA for 20 min, the adherent cells were stained by 0.1% crystal violet for 30 min. The adherent cell number was counted in four randomly selected x100 microscopic areas with phase contrast microscope.

### Colony formation assay

Colony formation assay was carried out as a previous study[26]. PC-3 cells were pre-treated by PAH (0, 0.25, 0.5, 1 μM) for 24 h before being plated onto 6-well plates and maintained in DMEM with 10% FBS for 12 days. Colonies were fixed by methanol for 10 min, stained by 1% crystal violet solution for 30 min, successively. The colony number was calculated in four randomly selected x100 fields. Plating efficiency = (number of colonies formed/number of cells inoculated) ×100%.

### Spheroid formation assay

Spheroid formation assay was conducted as a previous report[24]. After being pre-treated with PAH (0, 0.25, 0.5, 1 μM) for 24 h, PC-3 cells were plated onto 6-well plates, maintained in DMEM containing 20 μL/mL B27 (Invitrogen, Carlsbad, CA, USA), 10 ng/mL HCF (Sigma), 20 ng/mL epidermal growth factor (EGF, Sigma), 20 ng/mL basic fibroblast growth factor (bFGF, Invitrogen) and antibiotics for 2 weeks. The medium was replaced once every 2 days. Then, the number of prostaspheres with the diameter larger than 100 μm were counted in four randomly selected x100 areas with light microscope. Spheroid formation efficiency = average colonies/input cells×100%.

### Osteoclast differentiation assay

RAW264.7 cells were grown for 24 h, incubated with RANKL (100 μg/L) and PAH (0, 0.25, 0.5, 1 μM), then subjected to TRAP staining. The percentages of TRAP positive osteoclasts were quantified in three randomly selected x 100 fields by light microscope.

PC-3 cells were treated by PAH (0, 0.25, 0.5, 1 μM) for 24 h prior to the collection of the conditioned medium. Subsequently, RAW264.7 cells were treated by the aforementioned conditioned medium of PC-3 cells for 7 days, and then subjected to TRAP staining. The medium was replaced once every 2 days. The percentages of TRAP+ osteoclasts were counted from three randomly selected x100 fields with light microscope.

### Western blot analysis

PC-3 cells were pre-treated by PAH (0, 0.25, 0.5, 1 μM) for 14 days, afterward, western blot was performed with anti-CD133 and anti-CD44 antibodies.

PC-3 cells were pre-treated by PAH (0, 0.25, 0.5, 1 μM) for 24 h, thereafter, western blot was conducted with anti-RANKL antibody.

PC-3 cells were pretreated with PAH (0, 0.5, 1 μM) for 24 h, incubated with anti-RANKL antibody (0.5 mg/ml) or mouse IgG (0.5 mg/ml), prior to the collection of the conditioned medium. RAW264.7 cells were treated by the above conditioned medium of PC-3 cells for 7 days, and western blot was carried out with antibodies against IKKα, IKKβ, p65 and p50.

PC-3 cells were pretreated by PAH (0, 0.25, 0.5, 1 μM) for 24 h. Protein lysates were subjected to western blot analyses with antibodies against IKKα, IKKβ, subunits p65 and p50.

GAPDH was used as a loading control. The value from each group was further normalized with the loading control. The intensity of immunoreactivity was measured by Image J (Version 1.8.0; NIH).

### Statistical analysis

Data are expressed as mean ± standard deviation (SD). Student’s t-test or one-way ANOVA was used for the comparison of differences between two groups or among multiple groups. *P* values <0.05 indicated statistically significant.

## Results

### PAH inhibited the proliferation, invasion and migration of PC-3 cells

**Figure 1.**
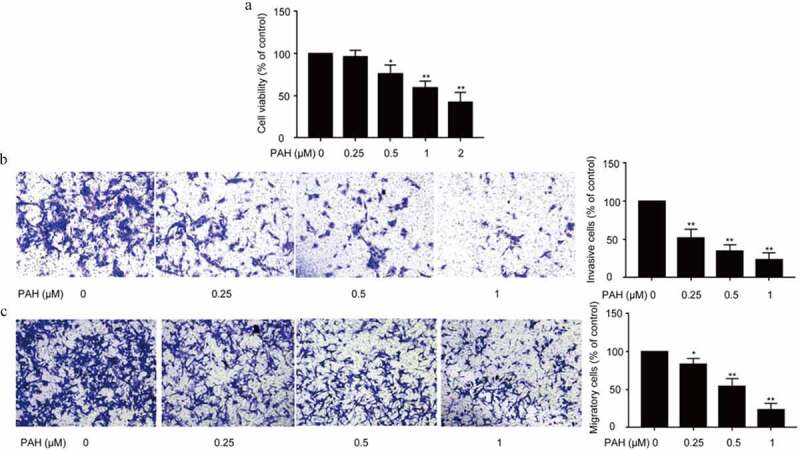
PAH inhibits the cell proliferation, invasion and migration of PC-3 cells dose-dependently. (a) PC-3 cells were treated with PAH (0, 0.25, 0.5, 1, 2 μM), and then subjected to CCK-8 proliferation assay. Five wells were assayed. (b) PC-3 cells were pretreated with PAH (0, 0.25, 0.5, 1 μM), and then subjected to Transwell invasion assays. The number of PC-3 cells which penetrated through the membrane was quantified in five randomly selected x100 microscopic areas. (c) PC-3 cells were pretreated with PAH (0, 0.25, 0.5, 1 μM), and then subjected to Transwell migration assays. The number of PC-3 cells which penetrated through the membrane was quantified in five randomly selected x100 microscopic areas. * p < 0.05, ** p < 0.01 vs. 0 μM of PAH. **Abrreviations**: PAH, perillaldehyde.

Cell proliferation, migration and invasion is essential for tumor growth and metastasis. Initially, the influence of PAH on cell proliferation of PC-3 cells was assessed with CCK-8. It was observed that PAH treatment (0.5, 1, 2 μM) resulted in a significant suppression on the PC-3 cell proliferation dose-dependently (Figure 1a).

Subsequently, the effects of PAH on the invasion and migration of PCa cells were examined with Transwell assay. Results exhibited that, PAH (0.25, 0.5, 1 μM) significantly suppressed the invasion and migration of PC-3 cells dose-dependently (Figure 1b,c).

### PAH prevented adhesion and colony formation of PC-3 cells

**Figure 2.**
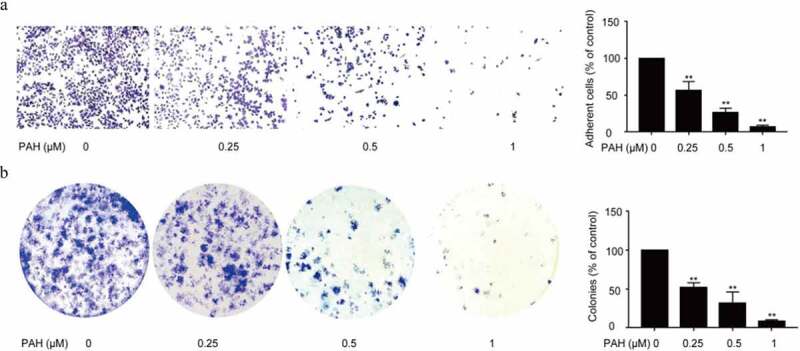
PAH prevents adhesion and adhesion-independent colony formation of PC-3 cells dose-dependently. (a) PC-3 cells were pretreated with PAH (0, 0.25, 0.5, 1 μM), and then subjected to cell-matrix adhesion assays. The number of PC-3 cells that had adhered to the 96-plate was quantified in four randomly selected x100 areas. (b) PC-3 cells were treated with PAH (0, 0.25, 0.5, 1 μM), and then subjected to colony formation assays. The number of colonies was counted in four randomly selected x100 fields. * p < 0.05, ** p < 0.01 vs. 0 μM of PAH. **Abrreviations**: PAH, perillaldehyde.

The metastasis of tumor cells from the primary tumor to distant other metastatic organs is a complex process involving adhesion and colony formation, in this study, we next examined if PAH could also prevent the cell-matrix mediated cell adhesion and colony formation of PCa cells. A significant decrease in the number of PC-3 cells pretreated with PAH (0.25, 0.5 and 1 μM) adhering to the baseline cell-matrix was observed, suggesting that PC-3 cell adhesion was repressed by PAH dose-dependently (Figure 2a).

Meanwhile, a remarkable decrease in the number of colonies was observed under PAH (0.25, 0.5 and 1 μM) pre-treated PC-3 cells, indicating that PAH inhibited the colony formation of PCa cells dose-dependently (Figure 2b).

### PAH prevented self-renewal capability of PC-3 cells

**Figure 3.**
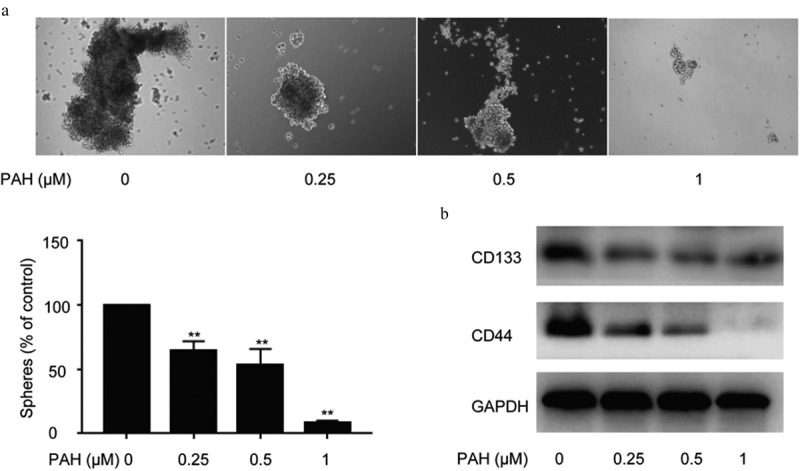
PAH inhibits spheroid formation and expression of CSC markers in PC-3 cells dose-dependently. (a) Spheroid formation of PC-3 cells pretreated with PAH (0, 0.25, 0.5, 1 μM). The number of Spheroids was quantified in four randomly selected x100 areas. (b) Protein lysates of PC-3 cells pretreated with PAH (0, 0.25, 0.5, 1 μM) were subjected to western blot analyses with CD133 and CD44 antibodies. * p < 0.05, ** p < 0.01 vs. 0 μM of PAH. **Abrreviations**: PAH, perillaldehyde.

Cancer stem cell hypothesis proposes that cancer are propagated by tumor cells with stem-characteristics,and result in tumor recurrence.To illustrate the function of PAH in the spheroid formation ability of PCa cells, spheroid formation assay of PC-3 cells pretreated with PAH was adapted. The results showed a concentration-dependent decrease in the number of prostaspheres after pretreatment of PAH (0.25, 0.5 and 1 μM) (Figure 3a). Furthermore, western blot analysis illustrated that PAH (0.25, 0.5 and 1 μM) repressed CD133 and CD44 expression, which are both CSC markers, in PC-3 cells dose-dependently (Figure 3b).

### PAH suppressed osteoclastogenesis induced by RANKL and PC-3 cells

**Figure 4.**
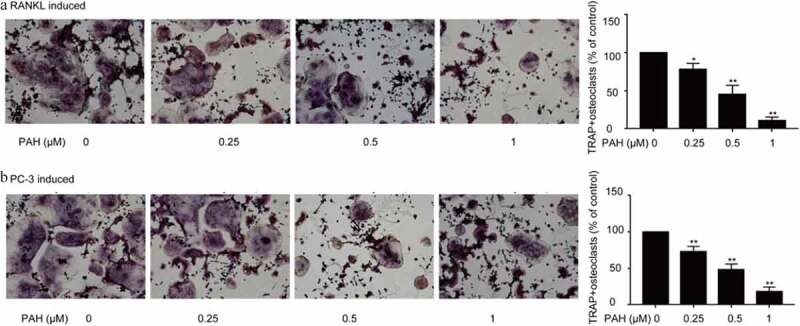
PAH arrests RANKL- and PC-3 cells-induced osteoclastogenesis dose-dependently. (a) RAW264.7 cells were cultured for 24 h, incubated with RANKL (100 μg/L) and different concentrations of PAH (0, 0.25, 0.5, 1 μM), then subjected to TRAP staining. The percentages of TRAP positive osteoclasts were quantified in three randomly selected x100 fields. (b) PC-3 cells were exposed to PAH (0, 0.25, 0.5, 1 μM) for 24 h, then the conditioned medium was collected. RAW264.7 cells were incubated with the aforementioned conditioned medium of PC-3 cells for 7 days, and then subjected to TRAP staining. The percentages of TRAP positive osteoclasts were quantified in three randomly selected x100 fields. * p < 0.05, ** p < 0.01 vs. 0 μM of PAH. **Abrreviations**: PAH, perillaldehyde; TRAP, tartrate resistant acid phosphatase; RANKL, receptor activator of nuclear factor-κB ligand.

In that study, we determined the RANKL-induced Osteoclastogenes in RAW264.7 cells by osteoclast differentiation assay.Osteoclastogenesis is usually associated with PCa by activating the RANKL signaling pathway [23,24]. As shown in Figure 4a, PAH (0.25, 0.5 and 1 μM) predominantly decreased RANKL-induced formation of osteoclasts in RAW264.7 cells dose-dependently. Whether PAH also inhibits PCa cells-induced osteoclastogenesis in RAW264.7 cells was further explored. PAH (0.25, 0.5 and 1 μM) significantly suppressed the PC-3 cells induced osteoclast differentiation in RAW264.7 cells dose-dependently (Figure 4b).

### PAH inhibited RANKL in PCa cells and inhibition of RANKL prevented PC-3 induced osteoclastogenesis

**Figure 5.**
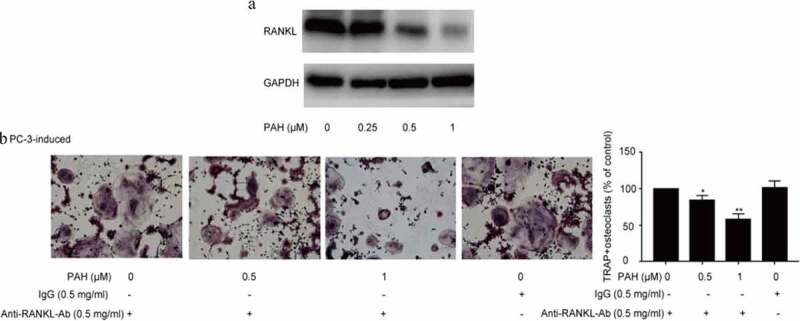
PAH inhibits RANKL expression and inhibition of RANKL suppresses PC-3 cells-induced osteoclastogenesis. (a) Protein lysates of PC-3 cells pretreated with PAH (0, 0.25, 0.5, 1 μM) were subjected to western blot analysis with RANKL antibody. (b) PC-3 cells were pretreated with PAH (0, 0.5, 1 μM) for 24 h, incubated with anti-RANKL antibody (0.5 mg/ml) or mouse IgG (0.5 mg/ml), then the conditioned medium was collected. RAW 264.7 cells were incubated with the above conditioned medium of PC-3 cells for 7 days, and finally subjected to TRAP staining. The percentages of TRAP positive osteoclasts were quantified in three randomly selected x100 fields. * p < 0.05, ** p < 0.01 vs. control. **Abrreviations**: PAH, perillaldehyde; TRAP, tartrate resistant acid phosphatase; RANKL, receptor activator of nuclear factor-κB ligand.

The activation of RANKL pathway prompts the activation of diverse downstream pathways required for osteoclast development.We further explored how PAH suppresses tumor cells induced osteoclastogenesis. Firstly, we evaluated if PAH could regulate RANKL expression in PC-3 cells. The results revealed that PC-3 cells indeed expressed RANKL, moreover, PAH (0.25, 0.5 and 1 μM) reduced RANKL expression dose-dependently (Figure 5a). Secondly, we detected the PCa cell-induced osteoclastogenesis in RAW264.7 cells at the condition of RANKL inhibition. It was observed that PC-3-induced osteoclastogenesis in RAW264.7 cells was significantly inhibited by PAH (0.5 and 1 μM) in a concentration-dependent manner, which was remarkably attenuated by inhibition of RANKL (Figure 5b).

### PAH suppressed RANKL-induced NF-κB activation via inhibiting IKK in PCa cells

**Figure 6.**
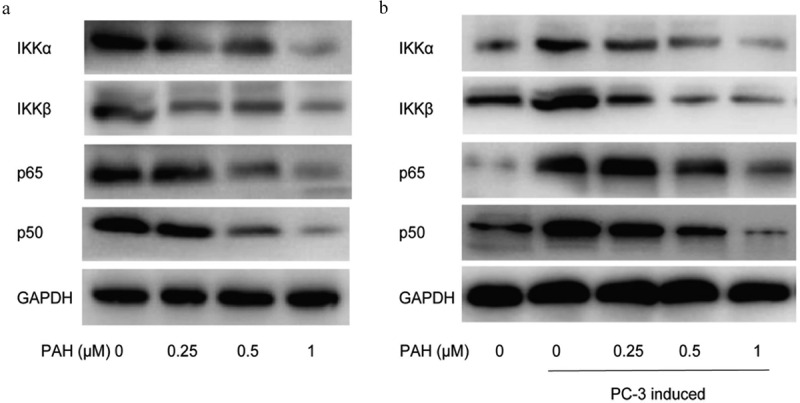
PAH inhibits PC-3 cell- and RANKL-induced activation of NF-κB via suppression of IKK dose-dependently. (a) Protein lysates of PC-3 cells pretreated with various concentrations of PAH (0, 0.25, 0.5, 1 μM) were subjected to western blot analyses with antibodies against mediators of the NF-κB pathway IKKα and IKKβ and NF-κB subunits p65 and p50. (b) PC-3 cells were treated with PAH (0, 0.25, 0.5, 1 μM) for 24 h, incubated with anti-RANKL antibody (0.5 mg/ml) or mouse IgG (0.5 mg/ml), and then the conditioned medium was collected. RAW264.7 cells were cultured in the above conditioned medium of PC-3 cells for 7 days, protein lysates of RAW264.7 cells were subjected to western blot analyses with IKKα and IKKβ, p65 and p50 antibodies. **Abrreviations**: PAH, perillaldehyde; IKK, inhibitor of nuclear factor kappa B (IκB) kinase.

Novel drugs that target RANKL pathway in osteoclasts could be effective treatments for bone metastases in PCa. To further illustrate the regulatory effect of PAH on osteoclastogenesis, we next examined the influence of PAH in NF-κB signaling pathway by western blot. The results demonstrated that PAH (0.25, 0.5 and 1 μM) reduced the expression of p65, p50, IKKα and IKKβ in PC-3 cells dose-dependently (Figure 6a). It implied that PAH inhibited PC-3 induced NF-κB activation via suppression of IKK. To explore the regulatory effect of PAH on RANKL-induced NF-κB activation, RAW264.7 cells were induced by PC-3 cells with the inhibition of RANKL. Expression of NF-κB signaling related markers of RAW264.7 cells was evaluated by western blot. Our data revealed that PAH (0.25, 0.5 and 1 μM) decreased the expression of p65, p50, IKKα and IKKβ in RAW264.7 cells dose-dependently (Figure 6b), illustrating that PAH significantly inhibited RANKL-induced NF-κB activation via suppression of IKK.

## Discussion

Prostate cancer has become a major public health concern worldwide. The interaction between prostate cancer cells and osteoblasts/osteoclasts can disrupt the normal dynamic balance of osteoblasts/osteoclasts and eventually lead to osteoblastic bone metastasis, which poses a great threat to human health. Therefore, it is of great interest to explore new treatments and drugs for prostate cancer. Naturally occurring compounds may be potential treatment options of metastatic PCa. For natural compounds, there have been some studies, for example, octanoic acid can promote the bone metastasis of PCA through the abnormal regulation of bone marrow adipo-osteogenic balance[27], and grape muscarus skin extract can also inhibit the generation of osteoclasts and alleviate bone metastasis[28]. Polyphenols play an important role in a variety of diseases, but there are few studies on their antitumor effects. Perillaldehyde, a kind of polyphenolic compounds, has many biological properties, including anticancer activity. However, the potential of PAH in inhibiting bone metastasis of prostate cancer has not been reported.

In the present study, we discovered that PAH treatments resulted in a suppression on the proliferation, invasion, migration, cell-matrix mediated cell adhesion capacity, and adhesion-independent cell proliferation of PC-3 cells dose-dependently, indicating the potential therapeutic role of PAH in metastatic PCa. PAH was found to decrease the number of tumor Spheroid and colonies in PC-3 cells as well as CD133 and CD44 expression of PC-3 cells, illustrating the essential function of PAH during the progression of PCa bone metastases by inhibiting the self-renewal ability and CSC protein expression of PCa cells.

To illustrate the suppressive effect of PAH on RANKL signaling- and PCa-induced osteoclastogenesis, RAW264.7 cell system was conducted. We found that PAH treatment decreased RANKL-induced formation of osteoclasts in RAW264.7 cells dose-dependently. Afterward, it was observed that PAH suppressed PC-3 induced osteoclast differentiation in RAW264.7 cells dose-dependently. It suggest that PAH inhibited osteoclast formation induced by RANKL and PCa cells in RAW264.7 cells. PC-3 induced osteoclastogenesis in RAW264.7 cells was significantly inhibited via PAH dose-dependently, which was patially attenuated by inhibition of RANKL, demonstrating that the effects of PAH was realized by targeting and decreasing RANKL. PAH exerts crucial suppressive effects on IKKα, IKKβ, p65 and p50 expression dose-dependently in PC-3 cells and PC-3 induced RAW264.7 cells. Taken together, our data strongly support that PAH suppresses RANKL- and PC-3 cells-induced osteoclastogenesis and activation of NF-κB signaling by blocking IKKα and IKKβ expression in PC-3 cells and PC-3 induced RAW264.7 cells.

As a natural product with anti-cancer property, it can be anticipated that PAH exerts the potential for the treatment of patients with metastatic PCa.

## Conclusions

Our study results demonstrated that PAH inhibited the tumor progression and stem cell characteristics of Prostate cancer, including proliferation, invasion and migration, colony formation and spheroid formation. Meanwhile, PAH suppressed cancer cell- induced osteoclastogenesis via activation the NF-κB pathway and receptor activator of nuclear factor-κB ligand.

## Data Availability

Data supporting the results reported in the manuscript can not be shared because they are related with our another ongoing study.

## Highlights

1. PAH suppressed proliferation, invasion and migration of PC-3 cells.

2.PAH prevented the stem cell characteristics of PC-3 cells, including cell-matrix adhesion, colony formation, spheroid formation as well as CD133 and CD44 expression.

3. PAH inhibited osteoclastogenesis.

4. PAH repressing the activation of NF-κB pathway as well as receptor activator of nuclear factor-κB ligand (RANKL)- and cancer cell- induced osteoclastogenesis in PCa cells.
